# 3′-Ferrocenylcarbon­yl-1′-methyl-4′-phenyl­spiro­[indeno­[2,3-*b*]quinoxaline-11,2′-pyrrolidine]

**DOI:** 10.1107/S1600536812042468

**Published:** 2012-10-20

**Authors:** B. Vijayakumar, D. Gavaskar, T. Srinivasan, R. Raghunathan, D. Velmurugan

**Affiliations:** a Centre of Advanced Study in Crystallography and Biophysics, University of Madras, Maraimalai (Guindy) Campus, Chennai 600 025, India; b Department of Organic Chemistry, University of Madras, Maraimalai (Guindy) Campus, Chennai 600 025, India

## Abstract

In the title compound, [Fe(C_5_H_5_)(C_31_H_24_N_3_O)], the pyrrolidine ring makes a dihedral angle of 86.3 (3)° with the mean plane [r.m.s deviation = 0.074 (2) Å] of the indeno-quinoxaline ring system. The central pyrrolidine ring adopts a twist conformation and the two cyclopentadienyl rings adopt an eclipsed conformation. In the crystal, mol­ecules are linked by weak C—H⋯N and C—H⋯π inter­actions, propagating along the *c* and *a* axes, respectively.

## Related literature

For the biological activity of ferrocene derivatives, see: Jaouen *et al.* (2004 ▶); Biot *et al.* (2004 ▶); Fouda *et al.* (2007 ▶). For related structures, see: Satis Kumar *et al.* (2007 ▶); Gunasekaran *et al.* (2010 ▶); Vijayakumar *et al.* (2012 ▶). For puckering and asymmetry parameters, see: Cremer & Pople (1975 ▶); Nardelli (1983 ▶).
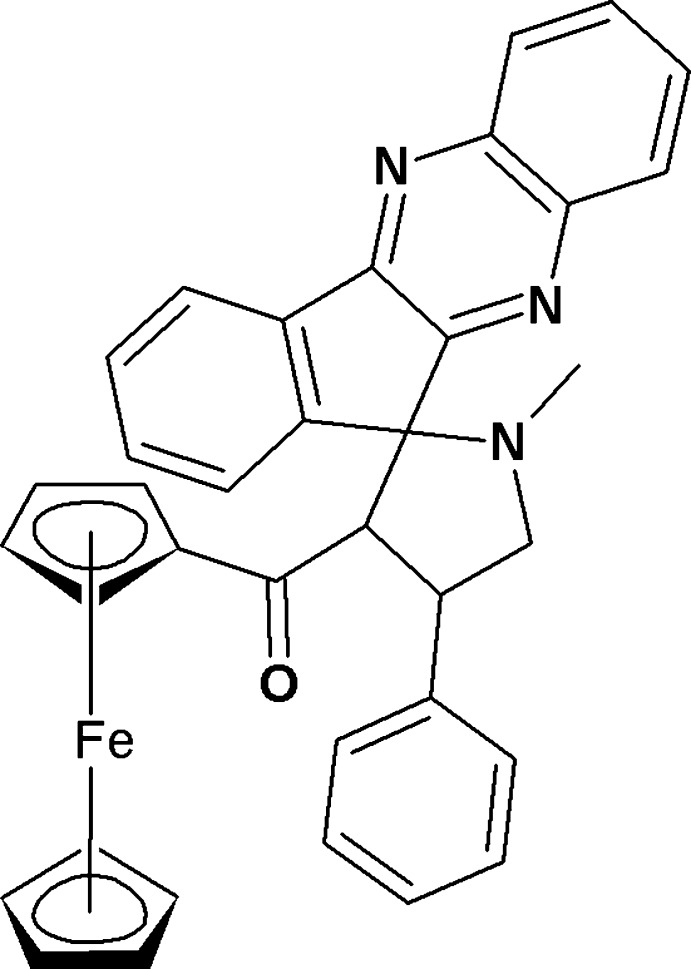


## Experimental

### 

#### Crystal data

[Fe(C_5_H_5_)(C_31_H_24_N_3_O)]
*M* *_r_* = 575.47
Monoclinic, 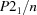

*a* = 11.1008 (4) Å
*b* = 11.9156 (5) Å
*c* = 21.0511 (9) Å
β = 90.944 (2)°
*V* = 2784.11 (19) Å^3^
*Z* = 4
Mo *K*α radiation
μ = 0.58 mm^−1^
*T* = 293 K
0.2 × 0.2 × 0.2 mm

#### Data collection

Bruker SMART APEX2 area-detector diffractometer
26663 measured reflections
7107 independent reflections
4920 reflections with *I* > 2σ(*I*)
*R*_int_ = 0.027

#### Refinement

*R*[*F* ^2^ > 2σ(*F* ^2^)] = 0.038
*wR*(*F* ^2^) = 0.105
*S* = 1.00
7107 reflections
370 parameters
H-atom parameters constrained
Δρ_max_ = 0.34 e Å^−3^
Δρ_min_ = −0.31 e Å^−3^

### 

Data collection: *APEX2* (Bruker, 2008 ▶); cell refinement: *SAINT* (Bruker, 2008 ▶); data reduction: *SAINT*; program(s) used to solve structure: *SHELXS97* (Sheldrick, 2008 ▶); program(s) used to refine structure: *SHELXL97* (Sheldrick, 2008 ▶); molecular graphics: *ORTEP-3* (Farrugia, 1997 ▶); software used to prepare material for publication: *SHELXL97* and *PLATON* (Spek, 2009 ▶).

## Supplementary Material

Crystal structure: contains datablock(s) global, I. DOI: 10.1107/S1600536812042468/lx2263sup1.cif

Structure factors: contains datablock(s) I. DOI: 10.1107/S1600536812042468/lx2263Isup3.hkl

Supplementary material file. DOI: 10.1107/S1600536812042468/lx2263Isup4.mol

Additional supplementary materials:  crystallographic information; 3D view; checkCIF report

## Figures and Tables

**Table 1:** Hydrogen-bond geometry (Å, °) *Cg*1 is the centroid of the C20–C25 ring.

*D*—H⋯*A*	*D*—H	H⋯*A*	*D*⋯*A*	*D*—H⋯*A*
C31—H31⋯N3^i^	0.98	2.45	3.430 (2)	176
C1—H1⋯*Cg*1^ii^	0.93	2.83	3.650 (2)	147

Symmetry codes: (i) 
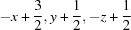
; (ii) 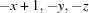.

**Fig. 1.**
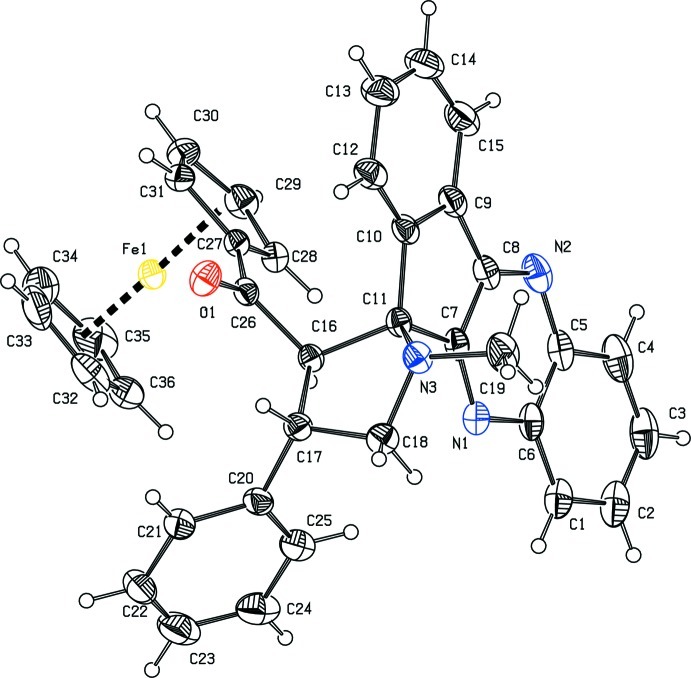
The molecular structure of the title compound with the atom numbering scheme. Displacement ellipsoids are drawn at the 30% probability level. H atoms are presented as small spheres of arbitrary radius.

**Fig. 2.**
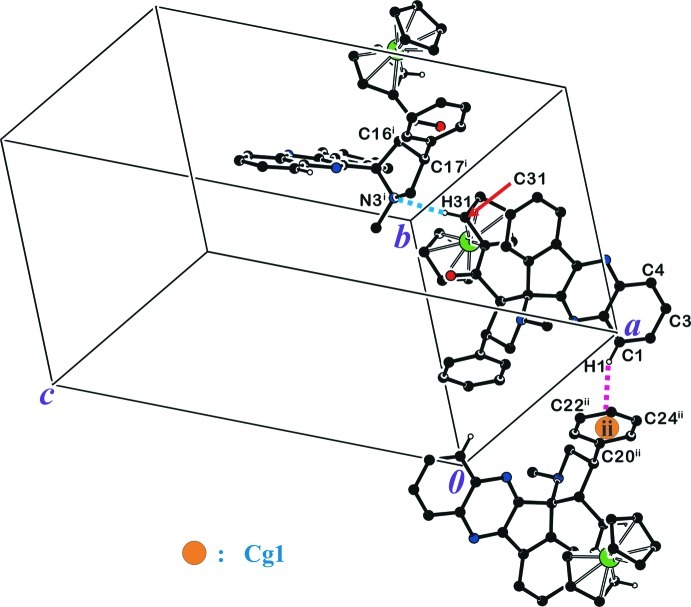
A view of the C—H···N and C—H···π interactions (dotted lines) in the crystal structure of the title compound. H atoms non-participating in hydrogen-bonding were omitted for clarity. [Symmetry codes: (i) -x + 3/2, y + 1/2, -z + 1/2; (ii) -x + 1, -y, -z.]

## supplementary crystallographic information

### Comment

Ferrocene derivatives are well known to have biological activities like antitumor (Jaouen *et al.*, 2004), antimalarial and antifungal (Biot *et al.*, 2004), and antibacterial (Fouda *et al.*, 2007). Against this background and in order to obtain detailed information on molecular conformations in the solid state, X-ray diffraction study of the title compound was carried out.

In the title compound (Fig. 1) the pyrrolidine ring adopts a twisted conformation with the puckering parameters q_2_ and φ and the smallest displacement asymmetric parameters, φ, as follows: q_2_ = 0.392 (2) Å, φ = 162.8 (3)° and Δ_2_(C16) = 1.84 (2)°. The indeno-quinoxaline unit is essentially planar, with a mean deviation of 0.074 (2) Å from the least-squares plane defined by the seventeen constituent atoms. The dihedral angle between the pyrrolidine ring (C11/C16–C18/N3) and the plane of indeno-quinoxaline fragment (C1–C6/N1/N2/C7–C15) is 86.3 (3)°. The Fe1···Cg2 and Fe1···Cg3 distances are 1.6461 (8) and 1.6506 (10) Å, respectively and the Cg2···Fe1···Cg3 angle is 177.91 (6)°, where Cg2 and Cg3 are the centroids of the C27–C31 and C32–C36 cyclopentadinene rings, respectively. In the crystal structure (Fig. 2), molecules are connected by weak C—H···N and C—H···π interactions (Table 1, Cg1 is the centroid of the C20–C25 phenyl ring).

### Experimental

A mixture of ninhydrin (1 mM) and 1,2-phenylenediamine (1 mM) was stirred with 10 ml of methanol for 10 min. To this mixture 1 mM of Sarcosine and 1 mM of ferrocene derived dipolarophile were added and was refluxed up to the end of the reaction as observed by TLC. The solvent content from the mixture was removed under reduced pressure and the crude product was obtained. Using column chromatography the crude extract was purified by 4:1 ratio of petroleum ether and ethyl acetate. Finally, single crystals suitable for the X-ray diffraction were obtained by slow evaporation at room temperature.

### Refinement

Hydrogen atoms were placed in calculated positions with C—H = 0.93Å and refined using the riding model approximation with a fixed isotropic displacement parameter of *U*_iso_(H) = 1.2*U*_eq_(C).

### Crystal data

**Table:**

[Fe(C_5_H_5_)(C_31_H_24_N_3_O)]	*F*(000) = 1200
*M**_r_* = 575.47	*D*_x_ = 1.373 Mg m^−^^3^
Monoclinic, *P*2_1_/*n*	Mo *K*α radiation, λ = 0.71073 Å
Hall symbol: -P 2yn	Cell parameters from 7107 reflections
*a* = 11.1008 (4) Å	θ = 1.9–28.7°
*b* = 11.9156 (5) Å	µ = 0.58 mm^−^^1^
*c* = 21.0511 (9) Å	*T* = 293 K
β = 90.944 (2)°	Block, brown
*V* = 2784.11 (19) Å^3^	0.2 × 0.2 × 0.2 mm
*Z* = 4	

### Data collection

**Table:**

Bruker SMART APEX2 area-detector diffractometer	4920 reflections with *I* > 2σ(*I*)
Radiation source: fine-focus sealed tube	*R*_int_ = 0.027
Graphite monochromator	θ_max_ = 28.7°, θ_min_ = 1.9°
ω and φ scans	*h* = −14→14
26663 measured reflections	*k* = −16→14
7107 independent reflections	*l* = −28→22

### Refinement

**Table:**

Refinement on *F*^2^	Primary atom site location: structure-invariant direct methods
Least-squares matrix: full	Secondary atom site location: difference Fourier map
*R*[*F*^2^ > 2σ(*F*^2^)] = 0.038	Hydrogen site location: difference Fourier map
*wR*(*F*^2^) = 0.105	H-atom parameters constrained
*S* = 1.00	*w* = 1/[σ^2^(*F*_o_^2^) + (0.0445*P*)^2^ + 1.0601*P*] where *P* = (*F*_o_^2^ + 2*F*_c_^2^)/3
7107 reflections	(Δ/σ)_max_ < 0.001
370 parameters	Δρ_max_ = 0.34 e Å^−^^3^
0 restraints	Δρ_min_ = −0.31 e Å^−^^3^

### Special details

**Table:**

Geometry. All esds (except the esd in the dihedral angle between two l.s. planes) are estimated using the full covariance matrix. The cell esds are taken into account individually in the estimation of esds in distances, angles and torsion angles; correlations between esds in cell parameters are only used when they are defined by crystal symmetry. An approximate (isotropic) treatment of cell esds is used for estimating esds involving l.s. planes.
Refinement. Refinement of F^2^ against ALL reflections. The weighted R-factor wR and goodness of fit S are based on F^2^, conventional R-factors R are based on F, with F set to zero for negative F^2^. The threshold expression of F^2^ > 2sigma(F^2^) is used only for calculating R-factors(gt) etc. and is not relevant to the choice of reflections for refinement. R-factors based on F^2^ are statistically about twice as large as those based on F, and R- factors based on ALL data will be even larger.

### Fractional atomic coordinates and isotropic or equivalent isotropic displacement parameters (Å^2^)

**Table:**

	*x*	*y*	*z*	*U*_iso_*/*U*_eq_	
C1	0.77481 (19)	0.11847 (19)	−0.10565 (10)	0.0523 (5)	
H1	0.7105	0.0704	−0.0983	0.063*	
C2	0.8184 (2)	0.1304 (2)	−0.16573 (10)	0.0639 (7)	
H2	0.7842	0.0896	−0.1991	0.077*	
C3	0.9136 (2)	0.2032 (2)	−0.17707 (11)	0.0683 (7)	
H3	0.9429	0.2099	−0.2180	0.082*	
C4	0.9646 (2)	0.2648 (2)	−0.12949 (11)	0.0654 (7)	
H4	1.0267	0.3146	−0.1383	0.079*	
C5	0.92353 (18)	0.25338 (18)	−0.06672 (10)	0.0493 (5)	
C6	0.82739 (17)	0.17886 (17)	−0.05517 (9)	0.0433 (4)	
C7	0.84103 (15)	0.21853 (15)	0.04944 (9)	0.0375 (4)	
C8	0.93787 (16)	0.29346 (16)	0.03778 (10)	0.0427 (4)	
C9	0.98033 (16)	0.33888 (16)	0.09867 (10)	0.0446 (5)	
C10	0.91387 (15)	0.28947 (16)	0.14705 (9)	0.0393 (4)	
C11	0.81387 (15)	0.21463 (15)	0.12047 (8)	0.0344 (4)	
C12	0.94162 (17)	0.31364 (18)	0.20968 (10)	0.0490 (5)	
H12	0.9004	0.2788	0.2423	0.059*	
C13	1.03199 (19)	0.3908 (2)	0.22318 (12)	0.0606 (6)	
H13	1.0509	0.4080	0.2653	0.073*	
C14	1.0946 (2)	0.4425 (2)	0.17492 (14)	0.0661 (7)	
H14	1.1533	0.4956	0.1849	0.079*	
C15	1.07068 (18)	0.41605 (19)	0.11241 (13)	0.0601 (6)	
H15	1.1142	0.4492	0.0800	0.072*	
C16	0.68110 (14)	0.25706 (14)	0.13186 (8)	0.0319 (4)	
H16	0.6470	0.2828	0.0912	0.038*	
C17	0.60983 (15)	0.15244 (15)	0.15325 (9)	0.0377 (4)	
H17	0.5996	0.1567	0.1993	0.045*	
C18	0.69421 (17)	0.05554 (16)	0.13916 (10)	0.0433 (4)	
H18A	0.6803	−0.0071	0.1675	0.052*	
H18B	0.6844	0.0303	0.0956	0.052*	
C19	0.91265 (19)	0.03021 (19)	0.13435 (12)	0.0611 (6)	
H19A	0.9875	0.0682	0.1428	0.092*	
H19B	0.9074	0.0106	0.0902	0.092*	
H19C	0.9089	−0.0367	0.1597	0.092*	
C20	0.48572 (16)	0.14804 (15)	0.12174 (10)	0.0422 (4)	
C21	0.38282 (18)	0.16230 (18)	0.15700 (12)	0.0574 (6)	
H21	0.3889	0.1662	0.2011	0.069*	
C22	0.2701 (2)	0.1709 (2)	0.12730 (16)	0.0743 (8)	
H22	0.2019	0.1822	0.1516	0.089*	
C23	0.2592 (2)	0.1628 (2)	0.06276 (17)	0.0775 (8)	
H23	0.1840	0.1694	0.0430	0.093*	
C24	0.3599 (2)	0.1448 (2)	0.02715 (13)	0.0665 (7)	
H24	0.3526	0.1372	−0.0167	0.080*	
C25	0.47266 (19)	0.13794 (18)	0.05648 (11)	0.0522 (5)	
H25	0.5404	0.1264	0.0319	0.063*	
C26	0.67864 (14)	0.35423 (15)	0.17847 (8)	0.0334 (4)	
C27	0.70406 (14)	0.46598 (15)	0.15256 (8)	0.0338 (4)	
C28	0.71680 (15)	0.49567 (16)	0.08699 (9)	0.0386 (4)	
H28	0.7184	0.4435	0.0510	0.046*	
C29	0.72666 (18)	0.61355 (18)	0.08356 (10)	0.0495 (5)	
H29	0.7344	0.6578	0.0446	0.059*	
C30	0.72074 (18)	0.65705 (17)	0.14583 (11)	0.0503 (5)	
H30	0.7234	0.7368	0.1572	0.060*	
C31	0.70684 (16)	0.56730 (16)	0.18877 (9)	0.0416 (4)	
H31	0.7005	0.5734	0.2350	0.050*	
C32	0.41038 (19)	0.4857 (3)	0.14284 (15)	0.0736 (8)	
H32	0.4028	0.4107	0.1615	0.088*	
C33	0.4164 (2)	0.5871 (4)	0.17646 (15)	0.0895 (10)	
H33	0.4130	0.5960	0.2227	0.107*	
C34	0.4264 (2)	0.6725 (3)	0.1322 (2)	0.0933 (11)	
H34	0.4319	0.7528	0.1421	0.112*	
C35	0.4259 (2)	0.6263 (3)	0.07184 (15)	0.0805 (8)	
H35	0.4313	0.6679	0.0318	0.097*	
C36	0.41605 (19)	0.5102 (2)	0.07812 (13)	0.0672 (7)	
H36	0.4137	0.4555	0.0433	0.081*	
N1	0.78365 (14)	0.16272 (13)	0.00513 (7)	0.0405 (4)	
N2	0.97974 (15)	0.31325 (16)	−0.01893 (9)	0.0536 (4)	
N3	0.81296 (13)	0.10361 (13)	0.15009 (7)	0.0407 (4)	
O1	0.65943 (13)	0.33960 (12)	0.23460 (6)	0.0482 (3)	
Fe1	0.56726 (2)	0.56669 (2)	0.124750 (13)	0.03939 (9)	

### Atomic displacement parameters (Å^2^)

**Table:**

	*U*^11^	*U*^22^	*U*^33^	*U*^12^	*U*^13^	*U*^23^
C1	0.0551 (12)	0.0581 (13)	0.0439 (12)	0.0195 (10)	0.0050 (9)	0.0000 (10)
C2	0.0678 (15)	0.0819 (18)	0.0420 (12)	0.0347 (14)	0.0023 (10)	−0.0004 (12)
C3	0.0685 (15)	0.0936 (19)	0.0435 (13)	0.0377 (14)	0.0174 (11)	0.0164 (13)
C4	0.0572 (13)	0.0820 (18)	0.0577 (15)	0.0201 (12)	0.0216 (11)	0.0225 (13)
C5	0.0432 (10)	0.0556 (13)	0.0497 (12)	0.0170 (9)	0.0140 (9)	0.0113 (10)
C6	0.0434 (10)	0.0452 (11)	0.0414 (10)	0.0182 (8)	0.0078 (8)	0.0061 (9)
C7	0.0330 (8)	0.0362 (10)	0.0434 (10)	0.0087 (7)	0.0077 (7)	0.0053 (8)
C8	0.0336 (9)	0.0404 (10)	0.0543 (12)	0.0059 (8)	0.0107 (8)	0.0096 (9)
C9	0.0306 (9)	0.0417 (11)	0.0615 (13)	0.0037 (8)	0.0045 (8)	0.0042 (9)
C10	0.0279 (8)	0.0385 (10)	0.0514 (11)	0.0056 (7)	−0.0009 (7)	0.0031 (8)
C11	0.0316 (8)	0.0339 (9)	0.0378 (9)	0.0045 (7)	0.0026 (7)	0.0028 (7)
C12	0.0374 (10)	0.0531 (12)	0.0564 (12)	0.0010 (9)	−0.0065 (9)	0.0032 (10)
C13	0.0466 (11)	0.0628 (14)	0.0717 (15)	−0.0017 (11)	−0.0159 (11)	−0.0051 (12)
C14	0.0420 (11)	0.0597 (15)	0.096 (2)	−0.0083 (10)	−0.0108 (12)	−0.0055 (14)
C15	0.0394 (11)	0.0545 (14)	0.0867 (18)	−0.0055 (9)	0.0071 (11)	0.0078 (12)
C16	0.0299 (8)	0.0332 (9)	0.0327 (9)	0.0012 (7)	0.0025 (7)	0.0034 (7)
C17	0.0371 (9)	0.0377 (10)	0.0385 (10)	−0.0044 (7)	0.0038 (7)	0.0013 (8)
C18	0.0472 (10)	0.0340 (10)	0.0488 (11)	−0.0009 (8)	0.0002 (8)	0.0074 (8)
C19	0.0513 (12)	0.0470 (12)	0.0850 (17)	0.0180 (10)	0.0033 (11)	0.0110 (12)
C20	0.0355 (9)	0.0340 (10)	0.0571 (12)	−0.0054 (7)	0.0025 (8)	−0.0009 (9)
C21	0.0443 (11)	0.0502 (13)	0.0781 (16)	−0.0082 (9)	0.0112 (11)	−0.0151 (11)
C22	0.0372 (11)	0.0623 (16)	0.124 (2)	−0.0028 (10)	0.0095 (13)	−0.0171 (16)
C23	0.0457 (13)	0.0585 (16)	0.128 (3)	−0.0015 (11)	−0.0218 (15)	0.0000 (16)
C24	0.0657 (15)	0.0541 (14)	0.0789 (17)	−0.0064 (11)	−0.0237 (13)	0.0039 (12)
C25	0.0477 (11)	0.0501 (12)	0.0586 (13)	−0.0039 (9)	−0.0048 (9)	0.0019 (10)
C26	0.0275 (8)	0.0391 (10)	0.0338 (9)	0.0038 (7)	0.0000 (6)	0.0002 (7)
C27	0.0288 (8)	0.0362 (9)	0.0362 (9)	0.0031 (7)	−0.0007 (7)	−0.0018 (7)
C28	0.0363 (9)	0.0404 (10)	0.0392 (10)	0.0027 (8)	0.0042 (7)	0.0004 (8)
C29	0.0471 (11)	0.0432 (11)	0.0585 (13)	−0.0021 (9)	0.0059 (9)	0.0116 (10)
C30	0.0491 (11)	0.0350 (10)	0.0669 (14)	−0.0044 (9)	0.0014 (10)	−0.0063 (10)
C31	0.0367 (9)	0.0420 (10)	0.0459 (11)	−0.0007 (8)	−0.0044 (8)	−0.0069 (9)
C32	0.0334 (11)	0.089 (2)	0.099 (2)	−0.0029 (12)	0.0050 (12)	0.0158 (17)
C33	0.0390 (12)	0.150 (3)	0.080 (2)	0.0184 (16)	0.0089 (12)	−0.033 (2)
C34	0.0580 (16)	0.074 (2)	0.147 (3)	0.0346 (14)	−0.0232 (18)	−0.034 (2)
C35	0.0606 (15)	0.086 (2)	0.094 (2)	0.0177 (14)	−0.0286 (14)	0.0182 (17)
C36	0.0403 (11)	0.0840 (19)	0.0768 (17)	0.0036 (11)	−0.0131 (11)	−0.0211 (15)
N1	0.0418 (8)	0.0402 (9)	0.0399 (9)	0.0067 (7)	0.0073 (7)	0.0024 (7)
N2	0.0442 (9)	0.0584 (11)	0.0588 (11)	0.0042 (8)	0.0167 (8)	0.0134 (9)
N3	0.0393 (8)	0.0342 (8)	0.0485 (9)	0.0050 (6)	0.0009 (7)	0.0080 (7)
O1	0.0615 (8)	0.0496 (8)	0.0336 (7)	0.0017 (7)	0.0078 (6)	0.0009 (6)
Fe1	0.03474 (14)	0.03809 (16)	0.04522 (17)	0.00631 (11)	−0.00323 (11)	−0.00167 (12)

### Geometric parameters (Å, º)

**Table:**

C1—C2	1.369 (3)	C20—C21	1.383 (3)
C1—C6	1.403 (3)	C20—C25	1.384 (3)
C1—H1	0.9300	C21—C22	1.393 (3)
C2—C3	1.392 (4)	C21—H21	0.9300
C2—H2	0.9300	C22—C23	1.365 (4)
C3—C4	1.358 (4)	C22—H22	0.9300
C3—H3	0.9300	C23—C24	1.373 (4)
C4—C5	1.412 (3)	C23—H23	0.9300
C4—H4	0.9300	C24—C25	1.389 (3)
C5—N2	1.375 (3)	C24—H24	0.9300
C5—C6	1.412 (3)	C25—H25	0.9300
C6—N1	1.380 (2)	C26—O1	1.217 (2)
C7—N1	1.303 (2)	C26—C27	1.468 (2)
C7—C8	1.422 (3)	C27—C31	1.428 (3)
C7—C11	1.531 (2)	C27—C28	1.434 (2)
C8—N2	1.310 (2)	C27—Fe1	2.0148 (16)
C8—C9	1.462 (3)	C28—C29	1.411 (3)
C9—C15	1.388 (3)	C28—Fe1	2.0364 (17)
C9—C10	1.397 (3)	C28—H28	0.9800
C10—C12	1.379 (3)	C29—C30	1.412 (3)
C10—C11	1.523 (2)	C29—Fe1	2.060 (2)
C11—N3	1.462 (2)	C29—H29	0.9800
C11—C16	1.580 (2)	C30—C31	1.410 (3)
C12—C13	1.387 (3)	C30—Fe1	2.058 (2)
C12—H12	0.9300	C30—H30	0.9800
C13—C14	1.385 (3)	C31—Fe1	2.0367 (18)
C13—H13	0.9300	C31—H31	0.9800
C14—C15	1.375 (3)	C32—C36	1.396 (4)
C14—H14	0.9300	C32—C33	1.401 (4)
C15—H15	0.9300	C32—Fe1	2.033 (2)
C16—C26	1.518 (2)	C32—H32	0.9800
C16—C17	1.547 (2)	C33—C34	1.385 (5)
C16—H16	0.9800	C33—Fe1	2.026 (2)
C17—C18	1.519 (3)	C33—H33	0.9800
C17—C20	1.520 (2)	C34—C35	1.386 (4)
C17—H17	0.9800	C34—Fe1	2.017 (2)
C18—N3	1.452 (2)	C34—H34	0.9800
C18—H18A	0.9700	C35—C36	1.395 (4)
C18—H18B	0.9700	C35—Fe1	2.037 (2)
C19—N3	1.453 (2)	C35—H35	0.9800
C19—H19A	0.9600	C36—Fe1	2.045 (2)
C19—H19B	0.9600	C36—H36	0.9800
C19—H19C	0.9600		
			
C2—C1—C6	119.9 (2)	C29—C28—H28	126.2
C2—C1—H1	120.1	C27—C28—H28	126.2
C6—C1—H1	120.1	Fe1—C28—H28	126.2
C1—C2—C3	120.3 (2)	C28—C29—C30	108.25 (18)
C1—C2—H2	119.9	C28—C29—Fe1	68.95 (11)
C3—C2—H2	119.9	C30—C29—Fe1	69.84 (11)
C4—C3—C2	121.3 (2)	C28—C29—H29	125.9
C4—C3—H3	119.4	C30—C29—H29	125.9
C2—C3—H3	119.4	Fe1—C29—H29	125.9
C3—C4—C5	120.1 (2)	C31—C30—C29	108.90 (18)
C3—C4—H4	120.0	C31—C30—Fe1	69.05 (11)
C5—C4—H4	120.0	C29—C30—Fe1	70.05 (11)
N2—C5—C4	119.0 (2)	C31—C30—H30	125.5
N2—C5—C6	122.38 (18)	C29—C30—H30	125.5
C4—C5—C6	118.6 (2)	Fe1—C30—H30	125.5
N1—C6—C1	118.49 (19)	C30—C31—C27	107.50 (17)
N1—C6—C5	121.62 (18)	C30—C31—Fe1	70.65 (11)
C1—C6—C5	119.89 (19)	C27—C31—Fe1	68.55 (9)
N1—C7—C8	123.99 (17)	C30—C31—H31	126.2
N1—C7—C11	125.57 (16)	C27—C31—H31	126.2
C8—C7—C11	110.44 (16)	Fe1—C31—H31	126.2
N2—C8—C7	123.44 (19)	C36—C32—C33	108.1 (3)
N2—C8—C9	128.20 (18)	C36—C32—Fe1	70.45 (14)
C7—C8—C9	108.35 (16)	C33—C32—Fe1	69.57 (15)
C15—C9—C10	121.0 (2)	C36—C32—H32	126.0
C15—C9—C8	130.5 (2)	C33—C32—H32	126.0
C10—C9—C8	108.45 (16)	Fe1—C32—H32	126.0
C12—C10—C9	119.76 (18)	C34—C33—C32	107.4 (3)
C12—C10—C11	128.55 (17)	C34—C33—Fe1	69.61 (16)
C9—C10—C11	111.67 (16)	C32—C33—Fe1	70.04 (14)
N3—C11—C10	112.52 (14)	C34—C33—H33	126.3
N3—C11—C7	116.54 (15)	C32—C33—H33	126.3
C10—C11—C7	100.77 (14)	Fe1—C33—H33	126.3
N3—C11—C16	102.23 (13)	C33—C34—C35	109.0 (3)
C10—C11—C16	115.65 (14)	C33—C34—Fe1	70.33 (15)
C7—C11—C16	109.73 (13)	C35—C34—Fe1	70.78 (15)
C10—C12—C13	118.9 (2)	C33—C34—H34	125.5
C10—C12—H12	120.5	C35—C34—H34	125.5
C13—C12—H12	120.5	Fe1—C34—H34	125.5
C14—C13—C12	121.0 (2)	C34—C35—C36	107.9 (3)
C14—C13—H13	119.5	C34—C35—Fe1	69.25 (14)
C12—C13—H13	119.5	C36—C35—Fe1	70.31 (13)
C15—C14—C13	120.6 (2)	C34—C35—H35	126.1
C15—C14—H14	119.7	C36—C35—H35	126.1
C13—C14—H14	119.7	Fe1—C35—H35	126.1
C14—C15—C9	118.6 (2)	C35—C36—C32	107.7 (3)
C14—C15—H15	120.7	C35—C36—Fe1	69.72 (14)
C9—C15—H15	120.7	C32—C36—Fe1	69.52 (13)
C26—C16—C17	114.31 (14)	C35—C36—H36	126.1
C26—C16—C11	111.65 (13)	C32—C36—H36	126.1
C17—C16—C11	105.60 (13)	Fe1—C36—H36	126.1
C26—C16—H16	108.4	C7—N1—C6	114.33 (16)
C17—C16—H16	108.4	C8—N2—C5	114.21 (18)
C11—C16—H16	108.4	C18—N3—C19	114.78 (16)
C18—C17—C20	116.49 (15)	C18—N3—C11	107.57 (13)
C18—C17—C16	103.63 (14)	C19—N3—C11	115.88 (16)
C20—C17—C16	111.41 (15)	C27—Fe1—C34	158.62 (13)
C18—C17—H17	108.3	C27—Fe1—C33	122.73 (12)
C20—C17—H17	108.3	C34—Fe1—C33	40.06 (13)
C16—C17—H17	108.3	C27—Fe1—C32	107.86 (10)
N3—C18—C17	103.31 (15)	C34—Fe1—C32	67.33 (13)
N3—C18—H18A	111.1	C33—Fe1—C32	40.39 (12)
C17—C18—H18A	111.1	C27—Fe1—C28	41.46 (7)
N3—C18—H18B	111.1	C34—Fe1—C28	158.17 (14)
C17—C18—H18B	111.1	C33—Fe1—C28	160.99 (13)
H18A—C18—H18B	109.1	C32—Fe1—C28	125.61 (10)
N3—C19—H19A	109.5	C27—Fe1—C31	41.26 (7)
N3—C19—H19B	109.5	C34—Fe1—C31	121.89 (11)
H19A—C19—H19B	109.5	C33—Fe1—C31	105.69 (10)
N3—C19—H19C	109.5	C32—Fe1—C31	121.51 (10)
H19A—C19—H19C	109.5	C28—Fe1—C31	69.12 (8)
H19B—C19—H19C	109.5	C27—Fe1—C35	159.50 (11)
C21—C20—C25	118.00 (19)	C34—Fe1—C35	39.97 (12)
C21—C20—C17	120.82 (19)	C33—Fe1—C35	67.43 (13)
C25—C20—C17	121.02 (17)	C32—Fe1—C35	67.27 (12)
C20—C21—C22	120.8 (2)	C28—Fe1—C35	123.85 (11)
C20—C21—H21	119.6	C31—Fe1—C35	158.63 (11)
C22—C21—H21	119.6	C27—Fe1—C36	123.52 (9)
C23—C22—C21	120.3 (2)	C34—Fe1—C36	67.19 (11)
C23—C22—H22	119.8	C33—Fe1—C36	67.57 (11)
C21—C22—H22	119.8	C32—Fe1—C36	40.04 (10)
C22—C23—C24	119.7 (2)	C28—Fe1—C36	110.11 (9)
C22—C23—H23	120.2	C31—Fe1—C36	158.19 (10)
C24—C23—H23	120.2	C35—Fe1—C36	39.97 (11)
C23—C24—C25	120.2 (2)	C27—Fe1—C30	68.38 (8)
C23—C24—H24	119.9	C34—Fe1—C30	107.22 (11)
C25—C24—H24	119.9	C33—Fe1—C30	120.65 (11)
C20—C25—C24	121.0 (2)	C32—Fe1—C30	156.49 (11)
C20—C25—H25	119.5	C28—Fe1—C30	67.93 (8)
C24—C25—H25	119.5	C31—Fe1—C30	40.29 (8)
O1—C26—C27	121.91 (16)	C35—Fe1—C30	124.26 (11)
O1—C26—C16	121.58 (16)	C36—Fe1—C30	161.12 (11)
C27—C26—C16	116.49 (14)	C27—Fe1—C29	68.61 (8)
C31—C27—C28	107.67 (16)	C34—Fe1—C29	122.39 (13)
C31—C27—C26	124.84 (16)	C33—Fe1—C29	156.40 (13)
C28—C27—C26	127.15 (16)	C32—Fe1—C29	162.17 (11)
C31—C27—Fe1	70.19 (10)	C28—Fe1—C29	40.28 (8)
C28—C27—Fe1	70.08 (10)	C31—Fe1—C29	68.17 (8)
C26—C27—Fe1	119.97 (11)	C35—Fe1—C29	109.54 (11)
C29—C28—C27	107.68 (17)	C36—Fe1—C29	126.24 (10)
C29—C28—Fe1	70.77 (11)	C30—Fe1—C29	40.11 (8)
C27—C28—Fe1	68.46 (9)		
			
C6—C1—C2—C3	−0.8 (3)	C26—C27—Fe1—C30	157.15 (16)
C1—C2—C3—C4	−0.7 (3)	C31—C27—Fe1—C29	80.85 (12)
C2—C3—C4—C5	1.8 (3)	C28—C27—Fe1—C29	−37.43 (11)
C3—C4—C5—N2	177.4 (2)	C26—C27—Fe1—C29	−159.60 (16)
C3—C4—C5—C6	−1.3 (3)	C33—C34—Fe1—C27	−42.9 (4)
C2—C1—C6—N1	−178.24 (18)	C35—C34—Fe1—C27	−162.2 (2)
C2—C1—C6—C5	1.2 (3)	C35—C34—Fe1—C33	−119.4 (3)
N2—C5—C6—N1	0.6 (3)	C33—C34—Fe1—C32	38.21 (18)
C4—C5—C6—N1	179.28 (18)	C35—C34—Fe1—C32	−81.2 (2)
N2—C5—C6—C1	−178.88 (18)	C33—C34—Fe1—C28	169.1 (2)
C4—C5—C6—C1	−0.2 (3)	C35—C34—Fe1—C28	49.7 (4)
N1—C7—C8—N2	−0.5 (3)	C33—C34—Fe1—C31	−75.8 (2)
C11—C7—C8—N2	−179.79 (17)	C35—C34—Fe1—C31	164.81 (17)
N1—C7—C8—C9	−179.20 (16)	C33—C34—Fe1—C35	119.4 (3)
C11—C7—C8—C9	1.5 (2)	C33—C34—Fe1—C36	81.8 (2)
N2—C8—C9—C15	2.7 (3)	C35—C34—Fe1—C36	−37.59 (19)
C7—C8—C9—C15	−178.7 (2)	C33—C34—Fe1—C30	−117.43 (19)
N2—C8—C9—C10	−176.40 (18)	C35—C34—Fe1—C30	123.20 (19)
C7—C8—C9—C10	2.2 (2)	C33—C34—Fe1—C29	−158.70 (17)
C15—C9—C10—C12	−2.9 (3)	C35—C34—Fe1—C29	81.9 (2)
C8—C9—C10—C12	176.24 (16)	C34—C33—Fe1—C27	162.85 (16)
C15—C9—C10—C11	175.70 (17)	C32—C33—Fe1—C27	−78.89 (19)
C8—C9—C10—C11	−5.1 (2)	C32—C33—Fe1—C34	118.3 (3)
C12—C10—C11—N3	−51.0 (2)	C34—C33—Fe1—C32	−118.3 (3)
C9—C10—C11—N3	130.52 (16)	C34—C33—Fe1—C28	−167.5 (3)
C12—C10—C11—C7	−175.86 (18)	C32—C33—Fe1—C28	−49.2 (4)
C9—C10—C11—C7	5.68 (18)	C34—C33—Fe1—C31	121.23 (18)
C12—C10—C11—C16	65.9 (2)	C32—C33—Fe1—C31	−120.50 (17)
C9—C10—C11—C16	−112.52 (17)	C34—C33—Fe1—C35	−37.32 (18)
N1—C7—C11—N3	54.5 (2)	C32—C33—Fe1—C35	80.95 (19)
C8—C7—C11—N3	−126.27 (16)	C34—C33—Fe1—C36	−80.7 (2)
N1—C7—C11—C10	176.52 (17)	C32—C33—Fe1—C36	37.53 (17)
C8—C7—C11—C10	−4.21 (18)	C34—C33—Fe1—C30	80.2 (2)
N1—C7—C11—C16	−61.1 (2)	C32—C33—Fe1—C30	−161.52 (16)
C8—C7—C11—C16	118.22 (16)	C34—C33—Fe1—C29	50.0 (3)
C9—C10—C12—C13	2.8 (3)	C32—C33—Fe1—C29	168.3 (2)
C11—C10—C12—C13	−175.51 (18)	C36—C32—Fe1—C27	−121.22 (16)
C10—C12—C13—C14	−0.5 (3)	C33—C32—Fe1—C27	119.86 (19)
C12—C13—C14—C15	−1.8 (4)	C36—C32—Fe1—C34	81.02 (19)
C13—C14—C15—C9	1.8 (3)	C33—C32—Fe1—C34	−37.90 (18)
C10—C9—C15—C14	0.6 (3)	C36—C32—Fe1—C33	118.9 (3)
C8—C9—C15—C14	−178.4 (2)	C36—C32—Fe1—C28	−78.75 (18)
N3—C11—C16—C26	111.69 (15)	C33—C32—Fe1—C28	162.33 (18)
C10—C11—C16—C26	−10.9 (2)	C36—C32—Fe1—C31	−164.43 (14)
C7—C11—C16—C26	−124.01 (15)	C33—C32—Fe1—C31	76.6 (2)
N3—C11—C16—C17	−13.10 (17)	C36—C32—Fe1—C35	37.53 (17)
C10—C11—C16—C17	−135.71 (15)	C33—C32—Fe1—C35	−81.4 (2)
C7—C11—C16—C17	111.19 (16)	C33—C32—Fe1—C36	−118.9 (3)
C26—C16—C17—C18	−135.03 (15)	C36—C32—Fe1—C30	162.1 (2)
C11—C16—C17—C18	−11.90 (17)	C33—C32—Fe1—C30	43.1 (3)
C26—C16—C17—C20	98.96 (17)	C36—C32—Fe1—C29	−45.7 (4)
C11—C16—C17—C20	−137.91 (15)	C33—C32—Fe1—C29	−164.6 (3)
C20—C17—C18—N3	155.72 (16)	C29—C28—Fe1—C27	−118.92 (16)
C16—C17—C18—N3	33.00 (18)	C29—C28—Fe1—C34	44.1 (3)
C18—C17—C20—C21	129.3 (2)	C27—C28—Fe1—C34	163.1 (3)
C16—C17—C20—C21	−112.1 (2)	C29—C28—Fe1—C33	−157.8 (3)
C18—C17—C20—C25	−55.5 (2)	C27—C28—Fe1—C33	−38.9 (3)
C16—C17—C20—C25	63.1 (2)	C29—C28—Fe1—C32	165.02 (14)
C25—C20—C21—C22	−2.6 (3)	C27—C28—Fe1—C32	−76.07 (14)
C17—C20—C21—C22	172.7 (2)	C29—C28—Fe1—C31	−80.48 (13)
C20—C21—C22—C23	1.5 (4)	C27—C28—Fe1—C31	38.44 (10)
C21—C22—C23—C24	0.7 (4)	C29—C28—Fe1—C35	80.30 (17)
C22—C23—C24—C25	−1.8 (4)	C27—C28—Fe1—C35	−160.79 (14)
C21—C20—C25—C24	1.6 (3)	C29—C28—Fe1—C36	122.81 (14)
C17—C20—C25—C24	−173.73 (19)	C27—C28—Fe1—C36	−118.28 (13)
C23—C24—C25—C20	0.6 (3)	C29—C28—Fe1—C30	−37.05 (12)
C17—C16—C26—O1	23.5 (2)	C27—C28—Fe1—C30	81.86 (12)
C11—C16—C26—O1	−96.32 (18)	C27—C28—Fe1—C29	118.92 (16)
C17—C16—C26—C27	−158.23 (14)	C30—C31—Fe1—C27	118.71 (16)
C11—C16—C26—C27	81.98 (17)	C30—C31—Fe1—C34	−78.78 (19)
O1—C26—C27—C31	−1.2 (3)	C27—C31—Fe1—C34	162.51 (17)
C16—C26—C27—C31	−179.50 (15)	C30—C31—Fe1—C33	−119.18 (17)
O1—C26—C27—C28	−173.65 (16)	C27—C31—Fe1—C33	122.10 (16)
C16—C26—C27—C28	8.1 (2)	C30—C31—Fe1—C32	−160.09 (14)
O1—C26—C27—Fe1	−86.89 (18)	C27—C31—Fe1—C32	81.19 (15)
C16—C26—C27—Fe1	94.82 (15)	C30—C31—Fe1—C28	80.10 (12)
C31—C27—C28—C29	−0.24 (19)	C27—C31—Fe1—C28	−38.62 (10)
C26—C27—C28—C29	173.25 (16)	C30—C31—Fe1—C35	−51.3 (3)
Fe1—C27—C28—C29	60.17 (13)	C27—C31—Fe1—C35	−170.0 (3)
C31—C27—C28—Fe1	−60.41 (12)	C30—C31—Fe1—C36	172.2 (2)
C26—C27—C28—Fe1	113.08 (16)	C27—C31—Fe1—C36	53.5 (3)
C27—C28—C29—C30	0.2 (2)	C27—C31—Fe1—C30	−118.71 (16)
Fe1—C28—C29—C30	58.96 (14)	C30—C31—Fe1—C29	36.71 (12)
C27—C28—C29—Fe1	−58.71 (12)	C27—C31—Fe1—C29	−82.00 (12)
C28—C29—C30—C31	−0.2 (2)	C34—C35—Fe1—C27	161.5 (3)
Fe1—C29—C30—C31	58.25 (14)	C36—C35—Fe1—C27	42.6 (4)
C28—C29—C30—Fe1	−58.40 (13)	C36—C35—Fe1—C34	−118.9 (3)
C29—C30—C31—C27	0.0 (2)	C34—C35—Fe1—C33	37.4 (2)
Fe1—C30—C31—C27	58.86 (12)	C36—C35—Fe1—C33	−81.5 (2)
C29—C30—C31—Fe1	−58.85 (14)	C34—C35—Fe1—C32	81.3 (2)
C28—C27—C31—C30	0.15 (19)	C36—C35—Fe1—C32	−37.59 (17)
C26—C27—C31—C30	−173.54 (16)	C34—C35—Fe1—C28	−160.03 (19)
Fe1—C27—C31—C30	−60.19 (13)	C36—C35—Fe1—C28	81.04 (19)
C28—C27—C31—Fe1	60.34 (11)	C34—C35—Fe1—C31	−37.6 (4)
C26—C27—C31—Fe1	−113.35 (16)	C36—C35—Fe1—C31	−156.5 (2)
C36—C32—C33—C34	−0.3 (3)	C34—C35—Fe1—C36	118.9 (3)
Fe1—C32—C33—C34	59.88 (18)	C34—C35—Fe1—C30	−75.2 (2)
C36—C32—C33—Fe1	−60.18 (16)	C36—C35—Fe1—C30	165.84 (15)
C32—C33—C34—C35	0.3 (3)	C34—C35—Fe1—C29	−117.5 (2)
Fe1—C33—C34—C35	60.48 (19)	C36—C35—Fe1—C29	123.59 (17)
C32—C33—C34—Fe1	−60.15 (17)	C35—C36—Fe1—C27	−163.49 (17)
C33—C34—C35—C36	−0.2 (3)	C32—C36—Fe1—C27	77.51 (18)
Fe1—C34—C35—C36	59.97 (17)	C35—C36—Fe1—C34	37.6 (2)
C33—C34—C35—Fe1	−60.20 (18)	C32—C36—Fe1—C34	−81.4 (2)
C34—C35—C36—C32	0.0 (3)	C35—C36—Fe1—C33	81.1 (2)
Fe1—C35—C36—C32	59.34 (16)	C32—C36—Fe1—C33	−37.85 (19)
C34—C35—C36—Fe1	−59.30 (18)	C35—C36—Fe1—C32	119.0 (2)
C33—C32—C36—C35	0.2 (3)	C35—C36—Fe1—C28	−119.12 (18)
Fe1—C32—C36—C35	−59.47 (17)	C32—C36—Fe1—C28	121.88 (17)
C33—C32—C36—Fe1	59.63 (16)	C35—C36—Fe1—C31	157.0 (2)
C8—C7—N1—C6	2.2 (2)	C32—C36—Fe1—C31	38.0 (3)
C11—C7—N1—C6	−178.60 (15)	C32—C36—Fe1—C35	−119.0 (2)
C1—C6—N1—C7	177.24 (17)	C35—C36—Fe1—C30	−38.7 (4)
C5—C6—N1—C7	−2.2 (2)	C32—C36—Fe1—C30	−157.7 (3)
C7—C8—N2—C5	−1.3 (3)	C35—C36—Fe1—C29	−76.8 (2)
C9—C8—N2—C5	177.16 (18)	C32—C36—Fe1—C29	164.24 (16)
C4—C5—N2—C8	−177.52 (18)	C31—C30—Fe1—C27	−38.48 (11)
C6—C5—N2—C8	1.2 (3)	C29—C30—Fe1—C27	82.04 (12)
C17—C18—N3—C19	−174.78 (16)	C31—C30—Fe1—C34	119.31 (17)
C17—C18—N3—C11	−44.20 (18)	C29—C30—Fe1—C34	−120.17 (17)
C10—C11—N3—C18	160.03 (15)	C31—C30—Fe1—C33	77.70 (18)
C7—C11—N3—C18	−84.29 (18)	C29—C30—Fe1—C33	−161.78 (17)
C16—C11—N3—C18	35.34 (17)	C31—C30—Fe1—C32	46.7 (3)
C10—C11—N3—C19	−70.0 (2)	C29—C30—Fe1—C32	167.2 (2)
C7—C11—N3—C19	45.7 (2)	C31—C30—Fe1—C28	−83.31 (12)
C16—C11—N3—C19	165.29 (16)	C29—C30—Fe1—C28	37.21 (12)
C31—C27—Fe1—C34	−44.4 (3)	C29—C30—Fe1—C31	120.52 (17)
C28—C27—Fe1—C34	−162.7 (3)	C31—C30—Fe1—C35	159.88 (15)
C26—C27—Fe1—C34	75.1 (3)	C29—C30—Fe1—C35	−79.60 (17)
C31—C27—Fe1—C33	−75.80 (16)	C31—C30—Fe1—C36	−171.1 (3)
C28—C27—Fe1—C33	165.92 (14)	C29—C30—Fe1—C36	−50.5 (3)
C26—C27—Fe1—C33	43.75 (19)	C31—C30—Fe1—C29	−120.52 (17)
C31—C27—Fe1—C32	−117.72 (14)	C28—C29—Fe1—C27	38.50 (11)
C28—C27—Fe1—C32	124.00 (13)	C30—C29—Fe1—C27	−81.42 (13)
C26—C27—Fe1—C32	1.83 (18)	C28—C29—Fe1—C34	−162.14 (15)
C31—C27—Fe1—C28	118.28 (15)	C30—C29—Fe1—C34	77.94 (17)
C26—C27—Fe1—C28	−122.17 (18)	C28—C29—Fe1—C33	162.1 (3)
C28—C27—Fe1—C31	−118.28 (15)	C30—C29—Fe1—C33	42.2 (3)
C26—C27—Fe1—C31	119.55 (18)	C28—C29—Fe1—C32	−43.3 (4)
C31—C27—Fe1—C35	169.6 (3)	C30—C29—Fe1—C32	−163.3 (3)
C28—C27—Fe1—C35	51.3 (3)	C30—C29—Fe1—C28	−119.91 (17)
C26—C27—Fe1—C35	−70.9 (3)	C28—C29—Fe1—C31	83.04 (12)
C31—C27—Fe1—C36	−159.01 (13)	C30—C29—Fe1—C31	−36.88 (12)
C28—C27—Fe1—C36	82.71 (14)	C28—C29—Fe1—C35	−119.70 (14)
C26—C27—Fe1—C36	−39.46 (18)	C30—C29—Fe1—C35	120.39 (15)
C31—C27—Fe1—C30	37.60 (11)	C28—C29—Fe1—C36	−78.13 (16)
C28—C27—Fe1—C30	−80.68 (12)	C30—C29—Fe1—C36	161.96 (14)

### Hydrogen-bond geometry (Å, º)

Cg1 is the centroid of the C20–C25 ring.

**Table:**

*D*—H···*A*	*D*—H	H···*A*	*D*···*A*	*D*—H···*A*
C31—H31···N3^i^	0.98	2.45	3.430 (2)	176
C1—H1···*Cg*1^ii^	0.93	2.83	3.650 (2)	147

Symmetry codes: (i) −*x*+3/2, *y*+1/2, −*z*+1/2; (ii) −*x*+1, −*y*, −*z*.
